# Prevalence, Clinical Correlates, and Use of Glucose-Lowering Drugs among Older Patients with Type 2 Diabetes Living in Long-Term Care Facilities

**DOI:** 10.1155/2015/174316

**Published:** 2015-09-06

**Authors:** Mario Bo, Stefano Gallo, Mauro Zanocchi, Paola Maina, Luisa Balcet, Martina Bonetto, Lorenzo Marchese, Annalisa Mastrapasqua, Nicoletta Aimonino Ricauda

**Affiliations:** ^1^Department of Medical Sciences, Geriatric Section, University of Turin, 10124 Turin, Italy; ^2^ASL TO4, 10034 Piemonte, Italy; ^3^Ospedale Civico “Città di Settimo Torinese”, 10036 Settimo Torinese, Italy

## Abstract

Prevalence, clinical correlates, and use of glucose-lowering drugs were comprehensively evaluated among 863 nursing home older patients with diabetes (mean age 82.9 ± 2.1 years): functional dependence and cognitive impairment were present in 84.1% and 68% of patients, respectively, and 66.3% of patients had 2–4 comorbidities. HbA1c values < 7.0% were documented in 54.9% of diabetic; significantly lower HbA1c levels were observed in demented patients than in nondemented subjects. Documented hypoglycemic episodes were reported for 57 patients (6.6%), without significant association with age, functional dependence, cognitive impairment, or HbA1c levels. About one-fifth of older long-term facilities residents have diabetes, with concomitant poor health conditions and high prevalence of cognitive impairment and functional dependence. Roughly three-fourths of these older and frail diabetic patients have HbA1c values lower than optimal, suggesting a potential for hypoglycemic harm especially among patients with severe cognitive impairment.

## 1. Introduction

There are few evidences about type 2 diabetes mellitus (T2DM) in frail elderly subjects and, specifically, in older patients living in long-term care residences [1–3]. These patients have usually reduced life expectancy, poor general health, and at least some degree of functional dependence and/or cognitive impairment. They represent those frail and vulnerable patients affected by T2DM for whom recent international guidelines specifically recommended less stringent glycemic targets and prioritized well-being and quality of life [4–6].

Duration of diabetes and advancing age independently predict morbidity and mortality rates in elderly subjects. Recent observations, demonstrating that cardiovascular complications and hypoglycemia are common among older diabetic patients, support the reorientation of care of older patients with T2DM away from intensive glycemic control as the core focus of management [7]. Target goal for glycated hemoglobin (HbA1c) in older adults generally should be 7.5% to 8%. Although HbA1c between 7% and 7.5% may be appropriate if it can be safely achieved in healthy older adults with few comorbidities and good functional status, higher HbA1c targets (8%-9%) are appropriate for older adults with multiple comorbidities, poor health, and limited life expectancy [4–6]. Moreover there is potential harm in lowering HbA1c to less than 6.5% in older adults with type 2 DM [8].

Despite these recommendations for older vulnerable diabetic patients, there is little evidence on prevalence, clinical correlates, and treatment of T2DM in elderly patients living in long-term care facilities. In the present study we aimed to comprehensively evaluate prevalence, clinical correlates, and use of glucose-lowering drugs among nursing home older patients with diabetes.

## 2. Materials and Methods

In this prospective observational study, patients living in 83 long-term care facilities in Piedmont, Northern Italy, were evaluated during the period of March–August 2013; all patients aged >65 and affected by diabetes were enrolled, without exclusion criteria.

Signed informed consent from patients or carer was obtained for all participants and the study was conducted according to the Recommendations Guiding Physicians in Biomedical Research Involving Human Subjects [9].

For all the patients the following information was recorded: identification, age, gender, and date of admission. Relevant conditions (as dementia, immobilization, and pressure sores) were also recorded. A thorough medical chart review was performed in order to ascertain, as far as possible, type and age of onset of diabetes, current hypoglycemic therapy, previous hypoglycemic episodes, and last available blood chemistries including serum glucose and glycated hemoglobin (HbA1c) levels and pre- and postprandial glucose levels. Total daily drug burden was also recorded.

Body mass index (BMI, according to the formula weight (kg)/height (m^2^)) was calculated and categorized in 4 classes (underweight: BMI < 18; normal weight: BMI 18–24.9; overweight: BMI 25–29.9; obesity: BMI ≥ 30). Standardized scales were used for the evaluation of functional autonomy and cognitive status. Functional status was evaluated using the Activities of Daily Living (ADL) scale [10] that measures six functions relating to activities necessary for self-care, in each of which the patient can be described as autonomous or dependent; a score equal to or higher than 2 identifies functional dependence. Cognitive status was evaluated using the Mini-Mental State Examination [11], a questionnaire evaluating several cognitive domains; score between 19 and 24/30 identifies mild cognitive impairment, while score between 10 and 18/30 and score below 10/30 identify moderate and severe cognitive impairment, respectively.

The data, collected on preprinted standardized protocols and subsequently transferred to MS Excel (Microsoft Inc.), were analyzed using SPSS/PC+. A preliminary explorative analysis was performed on continuous variables to assess normal distribution (skewness and kurtosis). The frequency of dichotomous and categorical variables was calculated, as well as the average and the standard deviation (SD) of continuous variables. Variables with Gaussian distribution were analyzed using Student's *t*-test and analysis of variance; variables without Gaussian distribution were analyzed using Pearson's Chi-square test. Dichotomous variables were analyzed using Chi-square test. The ANOVA test was used to evaluate differences between groups.

## 3. Results

Among 5076 residents in 83 long-term facilities, 863 patients with diabetes (17%, mean age 82.9 ± 2.1 years) were identified (Table 1), with female patients being significantly older than male patients (mean age 83.9 ± 1.9 versus 80.7 ± 2.2 years, *P* < 0.05). Among patients affected by diabetes there was a significantly greater prevalence of women than of men (582 women versus 281 men, 67.4% and 32.6% of diabetic patients, resp.) and this difference was statistically significant among patients aged 80 or more (*P* = 0.000). More than 97% of patients were ascertained to be affected by T2DM. Clinical documentation about age of onset of DM was retrievable only in 25.3% of patients: mean duration of T2DM in these patients was 12.9 ± 2.1 years at the moment of observation.

**Figure 1:**
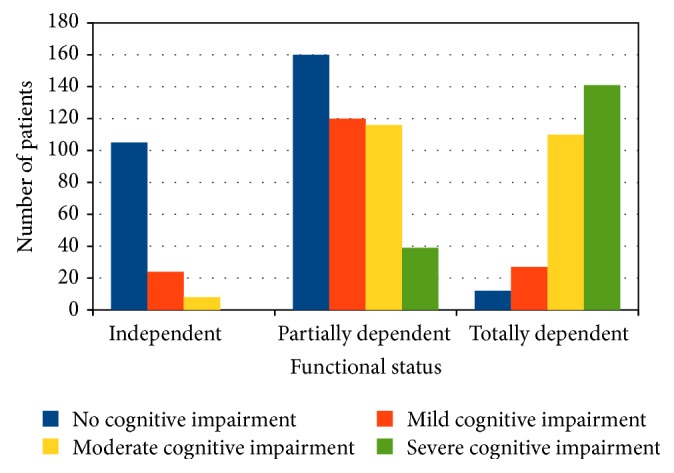
Distribution of cognitive impairment according to functional class among older diabetic patients.

It was possible to measure height and weight in 732 patients: roughly half of older diabetic patients were normal or underweight (44.3% and 5.3%, resp.), while a condition of overweight and obesity was documented in 23.9% and 11.4% of patients, respectively. Functional autonomy was documented in 15.9% of older diabetic patients, whereas partial or complete functional dependence was present in 50.4% and 33.7% of patients, respectively; 9.2% of patients were bedridden. Cognitive impairment was documented in 68% of diabetic patients, which was graded mild, moderate, and severe in 28.6%, 40.1%, and 31.3% of them, respectively. Coexistence of some degree of functional dependence and cognitive impairment was documented in 95.9% of patients (Figure 1). At least one comorbidity was observed in 99.2% of patients, with 66.3% of patients having 2–4 severe comorbidities. Hypertension (61.9%), dementia (36.8%), history of cardio- (22.8%) and cerebrovascular (21.3%) events, and bone fractures (13.4%) were the most common comorbidities observed.

At the moment of data collection, 14.4% of diabetic patients were not receiving hypoglycemic therapy, 41.3% were treated with oral hypoglycemic drugs, 35.3% were receiving insulin therapy, and 9.0% received combination therapy with oral hypoglycemic drugs and insulin. Among patients treated with oral hypoglycemic drugs, metformin was used by 61.5%, sulphonylureas by 33%, repaglinide by 9.3%, and a combination of metformin and glibenclamide by 4.8%; the remaining patients were treated with metformin and pioglitazone (0.8%), acarbose (0.8%), and metformin and DPP4 inhibitors (0.6%).

Among patients treated with insulin, 239 used rapid-acting insulin (25.4% human and 74.6% analogue), 58 were treated with intermediate-acting insulin, and 219 used long-acting (glargine or detemir) insulin (90.4% and 9.6%, resp.). Among patients treated with insulin therapy, 45.4% received 4 doses a day and 33.1% received 3 doses daily.

At least one value of glycated hemoglobin (HbA1c) was available in 74.6% of patients: 54.9% of patients had HbA1c values < 7.0%, 20.4% had HbA1c values between 7.0% and 8.5%, and 24.1% of patients had HbA1c values above 8.5%. In the total sample of older diabetic patients, no significant association was observed between classes of HbA1c level (<7.0%, 7.0–8.5%, and >8.5%) and either functional dependence or presence and severity of cognitive impairment, as well as mean HbA1c values, did not significantly differ in patients with or without functional dependence and cognitive impairment. Significantly lower HbA1c levels were observed in demented patients than in nondemented subjects (6.92 ± 1.28% versus 7.23 ± 1.67%, *P* = 0.013).

Preprandial glycemic values (available for 95.2% of patients) below 126 mg/dL were documented in 51.6% of patients; 26% of patients had values between 126 and 180 mg/dL and 17.7% had values above 180 mg/dL. Postprandial glycemic values under 180 mg/dL were observed in 37.2% of patients, 18.1% of patients had values between 181 and 250 mg/dL, and 8.1% of patients had values above 250 mg/dL. For 36.6% of patients there were no data available for postprandial glycemic values.

Documented hypoglycemic episodes were reported in medical charts for 57 (6.6%) patients. At the moment of data collection, 30 of these patients (52.6%) were treated with insulin, 22 (38.5%) received oral hypoglycemic agents (9 received metformin and glibenclamide, 2 received metformin and other sulphonylureas, 5 received metformin, 5 received sulphonylureas, and 1 received metformin and repaglinide), and 3 (5.2%) were treated with insulin and oral hypoglycemic agents (2 with acarbose and 1 with glibenclamide); 2 of these patients (3.5%) were not receiving drugs at the moment of data collection.

Among patients with reported previous hypoglycemic episodes 11 (19.3%) were bedridden and 19 (33.3%) were affected by severe cognitive impairment. At the moment of data collection, previous hypoglycemic episodes were not associated with age, functional dependence, or cognitive impairment, although a trend to a greater prevalence of hypoglycemia among demented patients was observed. Mean HbA1c levels were not significantly lower in patients with previous reported hypoglycemia than in other diabetic patients (6.83 ± 1.18 versus 7.143 ± 1.37, *P* = 0.08).

Single therapy with metformin was significantly more prevalent among patients without reported hypoglycemic episodes (*P* = 0.033). We did not observe significant association between use of sulphonylureas and hypoglycemic episodes, but therapy with metformin and glibenclamide was significantly more prevalent among patients with reported previous hypoglycemic episodes (*P* = 0.004). Patients receiving insulin therapy were significantly more prevalent among those with previous reported hypoglycemia (*P* = 0.009).

## 4. Discussion

We aimed to investigate prevalence, clinical correlates, and use of glucose-lowering drugs among older patients with T2DM living in long-term facilities in Piedmont, Northern Italy. Our study demonstrated that T2DM is a common clinical problem among these patients, affecting roughly less than one-fifth of residents, with a greater prevalence of the disease in women than in men. Extremely poor health conditions were documented in these patients. Less than one-fifth of them were functionally independent and roughly two-thirds of them had some degree of cognitive impairment, with concomitant functional dependence and cognitive impairment in more than 95% of patients. Two-thirds of patients had 2–4 severe comorbidities, mainly hypertension, dementia, cardio- and cerebrovascular disease, and bone fractures. Finally, we documented a high prevalence of low HbA1c values and a remarkable incidence of documented hypoglycemic episodes among these cognitively and functionally impaired older residents.

There are very few studies which investigated this item in similar populations. The prevalence of T2DM observed in our sample was in accordance with that observed in a French study, which reported a prevalence of T2DM of 17.1% among 6275 older long-term facility residents aged 86 years [12]. Because mean duration of T2DM in the patients enrolled in our study was around 12 years, it is reasonable to suppose that diabetes onset in most of the patients occurred probably at an older age.

Despite current recommendations on hypoglycemic treatment and HbA1c targets for older, frail, and vulnerable patients [4–6] we found that more than half of long-term facility older residents had HbA1c values below 7%, and 75% of them had levels below 8.5%. These findings demonstrate an undesired and potentially harmful aggressive hypoglycemic therapeutic approach in these frail and vulnerable patients. There are very few evidences about overall health benefit of hypoglycemic therapy among these frail patients, who are more vulnerable to and at higher risk of incident hypoglycemic episodes [8]. Therefore, recent international guidelines support the reorientation of care of older patients with T2DM away from intensive glycemic control as the core focus of management [7], and HbA1c targets around 8%-9% are deemed appropriate for older adults with multiple comorbidities, poor health, and limited life expectancy [4–6]. Moreover, HbA1c levels were significantly lower among demented patients, who are more prone to the negative consequences of hypoglycemia. There is a burden of evidence linking hypoglycemia and cognitive decline: cognitively impaired and demented patients are more prone and vulnerable to hypoglycemia, which itself represents a major risk factor for further cognitive decline [13–18]. Unawareness of hypoglycemia and subtle or atypical clinical presentation make extremely difficult an early diagnosis of hypoglycemia in demented, frail patients, leading to the potential for major harm in these patients.

Hypoglycemic episodes were reported in medical charts in 6.6% of patients, probably underestimating the true prevalence of this feared complication. Among patients with reported hypoglycemic events, roughly one-fifth of them were bedridden and one-third had severe cognitive impairment. At the moment of data collection HbA1c values below 7.5% were yet more frequent in patients with previous hypoglycemic episodes than in patients without prior hypoglycemic episodes (75.4% versus 45.1%, *P* = 0.03). Combination therapy with metformin and glibenclamide and insulin therapy were both significantly more frequent among patients with previous hypoglycemic episodes, whereas single therapy with metformin was more prevalent among T2DM patients without previous hypoglycemic episodes. These findings are in keeping with and reinforce current Beers recommendations about potentially inappropriate medication use in the elderly: metformin is considered the safest oral glucose-lowering approach in diabetic patients without specific contraindications, whereas rapid-acting insulins are considered the drugs with the highest potential for harmful hypoglycemic events [19]. However, because of the cross-sectional retrospective medical charts study, these findings should be carefully considered because we were able to document current glucose-lowering drug therapy at the moment of collection of data but we could not ascertain from medical charts which therapies were administered at the moment of hypoglycemic crisis.

Some limitations of the present study should be considered. The main limitation is inherent to the retrospective design of the study, based on data extracted from long-term facilities medical charts not scrupulously filled in. Moreover, retrospective observation makes it extremely difficult to define causality between adverse events and current hypoglycemic therapy, which however was not among the main goals of this study. On the other hand, this study in our view has some strengths that should be highlighted. To the best of our knowledge this is one of the first attempts to comprehensively evaluate global health status, including functional and cognitive conditions, among older patients with T2DM in long-term facilities. The high number of patients enrolled from a variegated sample of regional long-term facilities and the close similarity of our findings with results from the French study suggest that our results may reasonably and wisely be generalized to older Southern Europe patients living in long-term facilities.

In conclusion, our results documented that roughly three-fourths of older and frail diabetic patients living in long-term residences have HbA1c values lower than optimal, suggesting a potential for hypoglycemic harm especially among patients with severe cognitive impairment. Despite the current recommendations that strongly advise using “soft” HbA1c targets and wise and safe glucose-lowering medical therapies in these vulnerable patients, our findings seem to suggest an inappropriate and aggressive glucose-lowering therapeutic approach in most of these frail and vulnerable elderly residents.

## Figures and Tables

**Table 1:** Characteristics of diabetic patients living in long-term facilities.

Age (years)	
Total	82.9 ± 2.1
Male	80.7 ± 2.2
Female	83.9 ± 1.9
Females	582 (67.4%)
BMI	
≤18	46 (5.3%)
18–24.9	382 (44.3%)
25–29.9	206 (23.9%)
≥30	98 (11.4%)
N.D.	131 (15.1%)
Preprandial serum glucose	
≤70 mg/dL	47 (5.5%)
71–126 mg/dL	398 (46.1%)
127–180 mg/dL	224 (25.9%)
≥181 mg/dL	153 (17.7%)
N.D.	41 (4.8%)
HbA1c	
<7%	354 (54.9%)
7–8.5%	131 (20.4%)
>8.5%	159 (24.7%)
Functional dependence	
Partial (ADL = 1)	435 (50.4%)
Total (ADL ≥ 2)	291 (33.7%)
Moderate-severe cognitive impairment (MMSE ≤ 18)	616 (71.4%)
Comorbidities	
0	7 (0.8%)
1-2	261 (30.3%)
3-4	388 (44.9%)
≥5	207 (24.0%)
